# Making Space in Geographical Analysis

**DOI:** 10.1111/gean.12325

**Published:** 2022-03-23

**Authors:** Rachel S. Franklin, Elizabeth C. Delmelle, Clio Andris, Tao Cheng, Somayeh Dodge, Janet Franklin, Alison Heppenstall, Mei‐Po Kwan, WenWen Li, Sara McLafferty, Jennifer A. Miller, Darla K. Munroe, Trisalyn Nelson, Özge Öner, Denise Pumain, Kathleen Stewart, Daoqin Tong, Elizabeth A. Wentz

**Affiliations:** ^1^ 522468 Centre for Urban and Regional Development Studies (CURDS) School of Geography, Politics and Sociology Newcastle University Newcastle upon Tyne UK; ^2^ Alan Turing Institute for AI and Data Science The British Library London UK; ^3^ Department of Geography and Earth Sciences University of North Carolina at Charlotte Charlotte North Carolina USA; ^4^ School of City and Regional Planning School of Interactive Computing Georgia Institute of Technology Atlanta Georgia USA; ^5^ SpaceTimeLab Department of Civil Environmental and Geomatic Engineering University College London (UCL) London UK; ^6^ 8786 Department of Geography University of California Santa Barbara Santa Barbara California USA; ^7^ Department of Botany and Plant Sciences University of California Riverside California USA; ^8^ School of Political and Social Sciences MRC/CSO Social and Public Health Sciences Unit University of Glasgow Glasgow UK; ^9^ 26451 Department of Geography and Resource Management and Institute of Space and Earth Information Science The Chinese University of Hong Kong Hong Kong China; ^10^ 7864 School of Geographical Sciences and Urban Planning Arizona State University Tempe Arizona USA; ^11^ Department of Geography & Geographic Information Science University of Illinois Urbana‐Champaign Champaign Illinois USA; ^12^ Department of Geography and the Environment The University of Texas at Austin Austin Texas USA; ^13^ 2647 Department of Geography The Ohio State University Columbus Ohio USA; ^14^ 2152 Department of Land Economy University of Cambridge Cambridge UK; ^15^ University Paris I Pantheon Sorbonne and CNRS Paris France; ^16^ Department of Geographical Sciences University of Maryland College Park, Maryland USA

## Abstract

In this commentary we reflect on the potential and power of geographical analysis, as a set of methods, theoretical approaches, and perspectives, to increase our understanding of how space and place matter for *all*. We emphasize key aspects of the field, including accessibility, urban change, and spatial interaction and behavior, providing a high‐level research agenda that indicates a variety of gaps and routes for future research that will not only lead to more equitable and aware solutions to local and global challenges, but also innovative and novel research methods, concepts, and data. We close with a set of representation and inclusion challenges to our discipline, researchers, and publication outlets.

## Introduction

In the beginning, there was an isotropic plain—a boundless, featureless landscape—inhabited by a uniformly distributed population, all rational actors in possession of perfect information and with exactly the same preferences and needs. From these precepts the world of geographical analysis was born: theoretical frameworks, of course, but also methodological approaches and perspectives that embodied these assumptions. Geography and its cognate disciplines have since matured in their outlook: the landscape is not only *full* of features, but these features, whether physical geography, street networks, the built environment, or heterogeneous neighborhood contexts, are accepted as crucial and predictable determinants and constraints on behaviors, outcomes, and preferences. Where the population is concerned, rationality is no longer presumed and limits to knowledge are often understood as an integral element of decision‐making. Entire bodies of literature focus on the nature of non‐uniform population distribution. Regarding the preferences and needs of the population, however, the picture is cloudier. Certainly, individuals are not assumed to be uniform in wants, needs, or behaviors, and understanding the drivers of diverse outcomes and preferences has scarcely been ignored.

Nevertheless, the presumption of population homogeneity is in many ways written into our analytical DNA. Differences are something typically controlled for, or measured against a baseline that is, at best, representative of no group whatsoever and, at worst, representative mainly of people with access and power. Representation, or lack thereof, is a problem for our methods, our theory, and especially our capacity to respond to real‐world challenges. In this commentary, we take stock of how a more inclusive geographical analysis might look and offer some suggestions for how to get there.

On the one hand, spatial and geographical analyses are ideally placed to contribute to a more inclusive research agenda (and world). These approaches span a range of thematic areas such as transportation, health, environment, urban studies, and demography—with demonstrable connections to spatial and social inequality. Much of the research in these areas is applied and explicitly responds to vexing spatial problems around location, movement, interaction, and intersectionality. Moreover, ever‐increasing computational capacity, more rigorous methods, and expanding data availability should lend themselves to the articulated and nuanced requirements of analysis that addresses the particular needs of under‐represented and marginalized groups. On the other hand—how else to put it?—many of these challenges are not novel or emerging and still our field has yet to widely embrace the importance and potential of geographical analysis to elucidate and resolve spatial and geographical problems that center these groups and their lived experiences, preferences, and needs.

In the narrow universe of *Geographical Analysis*, just one journal of many in the field of spatial and geographical analysis, research explicitly about women, as just one example, has been rarely published in its 50 years of existence. Based on a search of the journal’s website, in well over 1,000 published pieces over the decades, fewer than 100 mention women anywhere in the body of the text. A similar number of articles contain the words “gender” or “female”. As might be expected, given the journal’s focus, many of these articles focus on migration and residential mobility, accessibility, health, and commuting. If article titles are any indication, although much of the research published in the journal is implicitly gendered, very little is *explicitly* about women and there has been an absence of papers that consider gender to be non‐binary. Only two papers contain the word “gender” in the title, by Tkocz and Kristensen (1994), on commuting, and Hoogstra (2012), on employment. Hanson and Kominiak (1997), writing on location and women’s labor market outcomes, is the only paper in 50 years that has “women” in the title. Similarly, there is only one paper with “female” in the title, on work and poverty (Kodras and Jones 1994). This is a shame, not only because so many areas of research in geographical analysis are clearly gendered and thus merit attention in their own right, but also because of the enormous research potential that exists, where methodological and technical innovations to address inequity are concerned.

Similar issues have been raised where science and the academic enterprise are concerned (Graves, Kearney, and Barabino 2022) and also within the broader discipline of geography, especially in relation to approaches like spatial analysis (Kwan 2004), and GIS (McLafferty 2005; Pavlovskaya and Martin 2007)—generally from a feminist geography perspective that highlights bigger questions around the social construction of knowledge, how knowledge is produced, and relationships between researcher and the researched (Staeheli and Lawson 1995). In the geography‐adjacent discipline of urban planning, gendered, or feminist, planning over the past several decades has emphasized the relevance of gender for both planning theory and practice. Sandercock and Forsyth (1992) enumerate the various spheres in which gender is key, including economic status, location and movement, and communication. Their examples—including childcare locations and public transportation scheduling—resonate in geographical and spatial analysis, as well. Feminist planning also draws attention to women’s “right to the city,” showing how default research and policy practices elide the needs and preferences of women (Beebeejaun 2017).

In geographical analysis, our claims or assumptions of neutrality and universality in data, methods, models, and applications have hampered our capacity to uncover (analytically and conceptually) the ways in which our research is gendered, age‐biased, color‐blind, or Global North‐centered. This in turn limits our ability to contribute to solutions to challenges that operate at the full range of spatial scales. As Staeheli and Lawson (1995, p. 333), writing in the pages of this journal, stated, “much work in this journal is used for management or planning purposes, but is not conducted with a goal of transforming social relations.” What they did not say, however, is that in order for research to contribute to effective management and planning, it must account for a diversity of preferences, behaviors, and needs—and this includes *everyone*. There is also a convincing argument to be made in favor of geographical analysis that emphatically aims to transform both social and spatial relations for the better.

This commentary shines light on ways in which geographical analysis has already contributed to inclusive research and understanding. We also ask where the untapped areas are for fruitful, interesting, innovative, and impactful spatial and geographical analysis. The authors are all established researchers and part of the *Geographical Analysis* editorial team. As a collective, we have identified below what we view as important contributions and constraints, as well as areas of potential research in spatial data, methods, and applications. By laying out this agenda in the pages of *Geographical Analysis* we signal that such research is welcomed at this journal.^1^

The remainder of our commentary is divided into four sections. We first tackle the conundrum of population heterogeneity—*the who*—and what it implies for inclusive geographical analysis. Next, we describe *the what*: how the foundations of geographical analysis, topics such as accessibility and land use modeling, can incorporate inclusivity. Following that, we discuss *the how*. This is not only the central role of data and why representativeness matters, but also the ability of geospatial analysis methods to generate knowledge and insights on activities, behaviors, and perspectives of diverse groups. Finally, we close with *the why*, offering several examples of a more inclusive geographical analysis research agenda, and providing some final conclusions, opportunities, and challenges to the field.

## The who

The genesis of geographical analysis presumed identical preferences and behaviors of the population. While this assumption is, of course, not a reflection of the real world, sorting out how inclusivity and population are related to geographical analysis is complex. Superficially, adapting to demographic non‐uniformity is merely a question of measurement and representation: do data, models, and methods disaggregate by demographic sub‐groups? Most demographic characteristics are, however, mutable and socially constructed. Those inhabiting the isotropic plain *choose* how to identify and this identification may change over time. Moreover, as D’Ignazio and Klein (2020) note, “what gets counted counts.” There are social choices embedded in the socio‐demographic characteristics we choose to measure; simply because we do not measure it, does not mean it does not exist. A good example of the complexity engendered by measuring, “who,” is gender identity. In geographical analysis, as discussed above, it can be rare for even a gender binary to be acknowledged. That is, even in a world reduced to only men and women, the women are still often invisible. Our understanding of gender identity and biological sex is rapidly evolving—a gender binary is hopelessly inadequate to capture gendered experiences—and our data and methods inevitably lag. This does not mean we should not aim to keep up.

That is not all, however. Many of our conceptual and theoretical frameworks (to do with, e.g., who migrates and why? How is an efficient route measured? How is space‐time accessibility quantified? Who lives where?) are based on universal rules and, imperfectly and shakily, assumptions of what is “normal.” This includes, but is not limited to, gender, and encompasses other intersecting, socially defined dimensions such as race, ethnicity, and (dis)ability. To a large extent, our conceptual frameworks remain anchored around traditional household structures and relationships. With changes to timing and preferences of household formation and increasing single‐person households and aging adults living independently in many countries, it is worth reassessing our understanding and assumptions around migration, mobility, accessibility, and residential choice, as these have spillover effects on all other aspects of geographical analysis.

## The what

Individuals are supported and constrained by the geographical and social contexts in which they are embedded, which can impact their education, health, and economic outcomes (Yabiku et al. 2017; Sturge et al. 2020; Wong and Shaw 2011). These geographic contexts represent the physical locations that a person experiences throughout the days, weeks, and months of their lives. Intuitively, contexts, or activity spaces, are largely defined by the locations of an individual’s home, work, schools and shopping destinations, as well as the transportation routes that are used to move between these frequently accessed spaces. Activity spaces vary in size, but documenting their shape and size is expensive (and potentially invasive). Instead, researchers often rely on generalized activity spaces that may be representative of few people’s actual, lived experiences. However, research suggests that the character of activity spaces matters—larger spaces support social mobility by providing access to opportunities and social connections. On the other hand, larger and more fragmented activity spaces can also be a burden for those who are most often responsible for both domestic activities and contributing to the family income, and also for those whose personal security and safety concerns may effectively limit activity spaces.

Individuals inhabit spaces, but they also interact. Differences across groups in social versus spatiotemporal interactions have been recently identified and could be relevant to a better understanding of how members of various groups interact across space and time. For example, Yang et al. (2016) showed that while both females and males exhibit strong same‐gender preferences in general, the tendency was stronger for females in a social context, while males had stronger same‐gender preferences in a spatial context. As novel technologies such as wearable sensors (see Read et al. 2012) become more ubiquitous, our ability to generate accurate and consistent metrics for quantifying spatiotemporal interactions has increased. This will allow further study to better understand differences in spatiotemporal interactions and how these differences might impact communication, innovation, and other phenomena.

## The how

The reality of geographical analysis is that it is dependent on data, conceptual frameworks, and quantitative methods, all of which have the power to exclude, whether by omission or commission.

### Data

The typical data employed in geographical analysis may be more diverse in source, but in fact less demographically representative than traditional survey or administrative data. Geographers make use of a variety of novel data: identifying patterns mined from social media (e.g., Jiang, Ma and Yin 2016); gathering new insights from crowdsourced data (e.g., Nelson et al. 2021); and analyzing mobility from location‐based smart‐phone apps (e.g., Dodge, 2021) ), and improving quality of life using real‐time data from smart city devices (e.g., Li and Batty 2020). We are increasingly able to generate a plethora of data on processes that previously defied measurement. It is an exciting time to be a spatial data scientist!

However, when data are not collected with a representative sample design it is important to consider who is missing. A concern with current trends in big spatial data is that the people missing are *already* underserved and that using these data will reinforce systemic privilege. As an example, crowdsourced tools, such as open street maps (OSM), are most often contributed by people with access to technology and the knowledge needed to use that technology. The perceptions of children, aging adults, people with disabilities, and people with lower socioeconomic status, including more people of color, are not equally represented in many crowdsourcing data sets. Similarly, cell‐phone based aggregate mobility indices which have been widely used for measuring the impact of the COVID‐19 pandemic and for enhancing epidemiological models and forecasts, as well as policy making, are not fully representative of all population segments (Noi and Rudolph 2022). Using big geographic data sets without careful consideration of who is represented and missing risks reinforcing systematic exclusion and discrimination.

The case of bicycling is illustrative of data sets that underrepresent women, children, and non‐white bicyclists (Nelson et al. 2021). It would be easy to use these data and have all the discoveries exclude many of the most underserved road users. Instead, recent research has turned attention to the interesting methodological challenge, and social solution, of correcting biased data when conducting analysis. Addressing issues of bias in data has made for intriguing research questions, including analytical solutions that allow us to use data with high resolution—in space, time, and attribution of crowdsourced data—by statistically integrating a sample of data that does represent all people (Roy et al. 2019).

The way we represent social livelihoods and social capital in geographic space is also subject to representation considerations. Social capital can be defined as the set of opportunities that result from social relations (Bathelt and Glückler 2005, p. 1547) and geographic social capital (GSC) can roughly be conceptualized as the spatialization of these opportunities (Debertin and Goetz 2013). Social capital surveys have the potential to capture a wider range of voices as they report on frequency of volunteering, trust of neighbors, and activity in attending events and belonging to organizations that yield social, emotional and sometimes monetary benefits. Examples include the Human Development in Chicago Neighborhoods (Earls et al. 2007) and the Social Capital Benchmark Community Survey (Harvard Kennedy School Saguaro Seminar, 2006).

Points of interest (POIs) can also foster social capital, but POIs used in geographic studies tend to emphasize some relationships over others. For instance, a prominent social capital index includes local fitness clubs, golf courses, religious institutions, sports clubs, and bowling alleys (Rupasingha, Goetz, and Freshwater 2006; King et al. 2019)—locations that might be viewed as more important to men than to other groups. Even the title of sociologist Robert Putnam’s 2001 requiem for social capital in the U.S., *Bowling Alone,* highlights the loss of the bowling alley—a male‐dominated pastime. A key misstep may be in neglecting the household and public spaces as locations for hosting events or community interaction. To improve this potential inequity, GSC studies should consider time use surveys that emphasize social interactions, and note the setting of the social interactions.

As a sub‐field we must address the lack of standard approaches in GIS and spatial analysis for documenting and correcting data bias. Geographers have a duty to evaluate if the research we do with big data is not just good but also right. When knowledge is created using data that excludes key populations it may do more harm than good. For example, in the bicycling case, we may exacerbate inequities of transportation access if we make decisions using data from people that already have good access (Nelson et al. 2021). Furthermore, as geographical analysis increasingly incorporates artificial intelligence (AI) methods, more transparent, trustworthy, and open datasets and GeoAI models will need to be developed, and cohesive collaborations between AI experts and domain experts will need to be fostered to objectively evaluate and test these models (Li 2020).

### Frameworks

The “bread and butter” of geographical analysis—where theory and method meet—is epitomized by accessibility and activity space (or context) research. Although spatial analysis extends well beyond the confines of these two areas, both serve as representative examples for how a more inclusive approach might generate interesting, novel findings while also increasing our understanding of the world.

There is no clear consensus on how to define activity spaces. Common approaches include buffered areas around specific locations and travel routes, kernel density, standard deviation ellipses, and Delaunay triangulation to encompass all spaces (Sherman et al. 2005). All approaches have advantages and disadvantages in the ways in which space is represented and therefore interpreted. Underestimating activity space can imply simple travel patterns or low mobility, while overestimating could imply that individuals and groups of individuals have more access to amenities and opportunities than they actually do in reality (Wong 2018). Furthermore, there is no clear method to provide attribute information within activity spaces. For example, the mode of transportation connecting destinations could provide opportunities or challenges depending on the broader context.

For its part, accessibility has long been a major research area in geography, transportation research, and urban planning. However, conventional placed‐based accessibility measures have limitations that render them particularly unsuitable for capturing sub‐group differences. For instance, these measures evaluate accessibility based on a single reference location such as a person’s home location or residential neighborhood, ignoring that many trips that contribute to individual accessibility are made in the context of the sequential unfolding of the person’s daily activities and trips. Further, placed‐based measures do not take into account the effect of space‐time constraints which may render many opportunities unreachable. This is especially problematic for individuals who face restrictive space‐time constraints due to activities whose location or timing cannot be changed easily (Kwan 2000). Previous research (e.g., Kwan 1998) has found that all conventional measures of accessibility suffer from an inherent bias because they ignore the space‐time complexities of people’s daily activities and trips and the role of space‐time constraints. These results have methodological implications for the study of access to jobs and urban opportunities. See, for example, Thomas (2010), who highlights the importance of individual characteristics, as well as transportation mode.

### Methods

Similarly, two contrasting methodological approaches—agent‐based modeling and spatial optimization—can help elucidate the risks of applying blanket‐level characteristics and preferences to groups in a modeling setting.

Individual‐based modeling approaches such as agent‐based modeling and microsimulation have grown in popularity over the past twenty years (Heppenstall et al. 2021). Their ability to simulate individuals, alongside their unique characteristics and behaviors, make them a natural metaphor for modeling and understanding the impacts of individual decisions within social and spatial systems (Crooks and Wise 2013; Sturley, Newing, and Heppenstall 2018). The recent COVID‐19 pandemic has brought agent‐based models to the forefront of the attention of researchers and policymakers alike with several models emerging that purport to simulate COVID‐specific scenarios, ranging from transmission risks amongst a population to easing of lockdown restrictions (e.g., Kano et al. 2021). These applications highlight the strength and flexibility of agent‐based models to create and represent heterogeneous populations. And when combined with high performance computing, simulation platforms can help to distangangle the effects of demographic and environmental components in spatio‐temporal trajectories (Cottineau and Chapron 2015; Pumain and Reuillon 2017). With a growing interest and research agenda towards creating “digital twins” of cities, agent‐based models are an obvious candidate for representing populations within these models, but do these individual‐based models capture and represent all demographics?

Akin to all other models, agent‐based models are an abstraction of reality. The individuals and rules within an agent‐based model are created through a mixture of data analysis, insight from the literature, and expert knowledge. A key assumption, however, is that our data are representative of all individuals within the model. For example, Kano et al. (2021) created an agent‐based model to explore the impact of COVID on economic activities; the agents, however, are created as equal—each agent represents a household, and they have equal amounts of money. This uniformity in how individuals are treated is typical of many agent‐based models; they fail to capitalize on existing, available data sources to improve upon these basic assumptions. As demonstrated elsewhere in this commentary, there is a wealth of information and data that capture population diversity that can be embedded within these models, but to date they remain largely absent. If approaches such as agent‐based models are to be used to represent and simulate individuals and their behaviors in models that create and tailor policy, ensuring equitable representation must be a priority.

Spatial optimization concerns the identification of the best strategies to arrange entities, resources, or goods in space in order to achieve a single or multiple goals (Tong and Murray 2012). These techniques have been used to solve a range of real‐world problems, including political redistricting, land use planning, service facility placement, and bus route planning. In many spatial optimization models, common goals have been to achieve the maximal benefits/profits (e.g., accessibility to amenities) or minimize the overall cost (e.g., travel distance/time). When evaluating these goals, the population of interest is often aggregated using census data collection/summary units, such as U.S. Census block groups and tracts. This can be problematic as benefits/costs evaluation may vary among individuals based on their socio‐demographic characteristics.

As an example, when constructing a spatial optimization model to site public infrastructure such as libraries, the goal is often to maximize the accessibility of the libraries to residents in an area. Accessibility maximization is often achieved by minimizing the overall travel distance to the nearest library. However, considering that activity spaces may vary substantially among individuals, the library with the least travel distance, or time from home, may not represent the most accessible one. For example, a more convenient library might be on the way to another travel destination according to an individual’s daily travel program. A study by Li and Tong (2017) incorporated individual‐level activity spaces into the accessibility assessment when alternative service facilities are evaluated for siting. There is a clear need for new spatial optimization models to be developed to address these individual‐level differences.

## The why

If data are equated with power (Illiadis and Russo 2016)—the power to dictate research agendas, the questions being asked, and the variables included—then the implications of women, individuals of color, and other groups being disproportionately omitted from data sources serves to reinforce and deepen divisions. Similar challenges hold not only where methods and conceptual frameworks are concerned, but also in terms of substantive research areas. Below we highlight a selection of research areas where we see particular potential for an inclusive geographical analytical approach to generate novel insights and knowledge.

### Urban change

With respect to urban change research, new forms of smart human sensor data are correlated with location and income, concentrated in areas receiving the largest amount of attention in the form of capital investment and reinvestment. This skews research towards these areas, as work on gentrification—its causes and consequences—has far exceeded research on the disinvested or neglected urban and suburban places. Single women with children are the most probable group to be displaced when housing prices rise in gentrifying places (Curran 2017), but our research overwhelmingly *ends* at these origins. What about their destinations? What challenges (or opportunities) exist as the most vulnerable are pushed towards the older suburbs with precarious housing structures, disconnected streets, and spatial separation from most urban or newer suburban activities? Quantitatively capturing the experiences of the displaced has long confounded researchers (Easton, Lees and Hubbard 2020) and the convenience of new “big” data sources threatens to push this subject aside as we follow capital, investment, and the data it produces.

A research agenda on urban change that prioritizes issues that affect women and other under‐represented groups most acutely may include: understanding housing challenges faced by the population most likely to face risks of eviction—Black women with at least one child living near the poverty level (Curran 2017); addressing daily activity struggles for two‐working‐adult households with children in a landscape of segregated land‐uses and housing types (or a lack of family‐friendly housing in highly accessible urban spaces) (Moos 2016); and retrofitting older, suburban neighborhoods to meet the needs of their poor and minority residents, especially single‐parent females, who increasingly reside in these places (Hanlon and Airgood‐Obrycki 2018). These topics, among many others, will require creative solutions to filling in their data gaps, to peek into the shadows of big data so that knowledge may be produced about neglected urban spaces and their residents.

### Entrepreneurship

Today it is widely accepted that entrepreneurship is an essential tool for economic growth and development both in developed and developing parts of the world (Acs and Desai 2008). A significant and established body of literature investigates the determinants of entrepreneurship and self‐employment, emphasizing the importance of spatial factors, such as formal and informal local institutions, local labor market conditions and industrial composition, and local infrastructure. A parallel line of literature, on the other hand, looks at the specific conditions related to female entrepreneurship (e.g., Minnitti 2010; Estrin and Mickiewicz 2011; Jennings and Brush 2013). There exists, however, a lack of connection between these two strands of literature even though methodologically they are similar to one another.

The pecuniary and non‐pecuniary determinants of entrepreneurship, as well as its economic and well‐being consequences, are distinctly different for female entrepreneurs and the same is likely to be true for other groups not typically studied in the entrepreneurship literature. Existing work on the geography of entrepreneurship is quite mature in terms of the empirical designs and methods used. However, unfortunately, when it comes to understanding the role of gender there is a tradition of differentiating between female and male entrepreneurs in a binary fashion in analyses, by only controlling for gender, for example, or in the best case via some multivariate analysis for the female sub‐population. There is, however, sufficient evidence that the spatial contextual determinants and consequences of entrepreneurship and self‐employment are demonstrably different for women (Abreu, Oner, and Brouwer 2019), which may imply significant flaws with the gender‐neutral empirical designs and variable selections. There is ample room in the geographical analysis of entrepreneurship to explicitly consider the group‐specific determinants and consequences of entrepreneurship, such as availability of individual and local resources, the importance of networks and other peer‐groups (e.g., ethnic enclaves), and potential health and well‐being effects associated with entrepreneurship and self‐employment.

### Equitable mobility and spaces

Human movement is driven by its need to access resources and its social behavior. Movement patterns are also influenced to various degrees by people’s lifestyles, feelings, internal and external conditions across different individuals, and depending on their personal and household circumstances (Dodge 2021). Normally, individuals avoid certain locations, and their movements tend to cluster in their common daily activity space, with the exception of a few Lévy type longer distance trips (González, Hidalgo, and Barabási 2008). However, the “inter‐subjective” (Harari 2014) perception of risk in a population segment can also influence their movement decisions (e.g., travel distances, transport modes, time allocation, activities). The desire to access opportunities and resources and the fear of risk caused by, for example, crimes or pandemics can vary for different segments of the population. These can also change in different places and over time. To better understand vulnerable populations and implement more equitable policies for resource allocation, infrastructure planning, and mitigation measures, new models are needed to quantify human behavior by taking these parameters into account.

We define an “equitable space” as a multidimensional, multiscale space‐time landscape that represents equal access to opportunity, wellbeing, and spatial and temporal resources, as well as protection from crises or risk exposure for different population segments. This landscape is dynamic in a way that changes in response to normal conditions or a crisis situation such as a pandemic. It is also sensitive to demographic variables and the geographic structure of communities. The fundamental addition to traditional spatiotemporal models of human behavior (Hägerstrand 1970; Miller 1991; Miller, Dodge, and Miller 2019) is that people’s perception of risk or demands change their mobility behavior, personal space and time, and their reach of activity locations. The influence of risk perception and various levels of demands on human activity space may also be amplified in the time of crisis.

One way to address existing limitations is through the use of AI‐enabled geographical analysis—GeoAI—that enables a powerful, predictive framework capitalizing on a wide range of geo‐tagged and time‐stamped data. Some state‐of‐the‐art prediction algorithms have been developed to tackle issues specifically oriented toward network‐constrained events. These include network‐based crime hotspot predictive mapping (Rosser et al. 2017), street‐network‐based police patrolling algorithms (Chen and Cheng 2018), and a network‐based graph deep learning algorithm for sparse events (Zhang and Cheng 2020). These techniques offer some innovative developments for improving equitable utilization of space.

### Health and healthcare

Access to health care is strongly shaped by the spatial contexts in which individuals live, work, move, and interact. Inequalities at home, work, and in other spheres of daily life affect exposure to environmental hazards and benefits, access to services and resources, opportunities for social interaction, and everyday stresses and space‐time constraints that in turn influence physical, social, and emotional health and wellbeing. Gender, in particular, plays a critical role in healthcare access: women are often the primary procurers of healthcare for their families, and their access to quality health services, transportation, and health insurance, and their interactions with healthcare providers reflect gender‐related constraints and inequalities. Access challenges for the transgender and non‐binary communities have also been well documented.

Geographical analysis methods provide essential tools for understanding the dynamic and complex pathways linking an individual’s health and spatial contexts. These methods have been widely employed in investigating a range of health issues. However, most of these studies simply consider gender as another predictor variable while neglecting dynamic and gender‐specific interactions between spatial contexts and health and healthcare outcomes. The spatiotemporal characteristics of geographic contexts relevant for health differ across people, calling for person‐based measures that incorporate gender constraints and disparities (Kwan 2012). Attention to intersectionality is also critically important, as geographic contexts are shaped by interlocking axes of social difference including race, ethnicity, class, and gender. Moreover, geographic analyses rarely address individual‐level perceptions and experiences of space that affect stress, mobility, and psychosocial wellbeing. Incorporating these important issues in geographic modeling requires place‐specific and context‐aware approaches that consider dynamic interactions between people and places (McLafferty 2020).

### Mobility and health

During the time of the COVID‐19 pandemic, increased attention has been given to changes in mobility including a major reduction in both local and long‐distance travel, as well as the role of mobility in controlling the spread of this highly infectious disease on the one hand (Zhou et al. 2020), or contributing to spread on the other (Chang et al. 2020; Giles et al. 2021). From personal social interactions and daily movements, to movements over larger spatial extents including travel between countries, researchers have examined mobility trends during major events such as the pandemic (Kang et al. 2020; Lee et al. 2020; Fan and Stewart 2021; Kim and Kwan 2021). This holds, of course, not only for COVID‐19, but for other infectious diseases, such as malaria, where patterns of movement have been studied particularly with respect to risk of transmission (Sinha et al. 2020; Wesolowski et al. 2012).

The investigation of mobility and its relationship with different factors relating to infectious diseases offers numerous opportunities for research, including a closer analysis of mobility and individual‐level characteristics. This could involve, for example, analyzing the movements of women, or other under‐represented groups, to understand the impact of a pandemic on their unique mobility characteristics, and whether their patterns of movement leave them vulnerable to exposure to an infectious disease. In analyses of mobility by occupation in regions plagued by infectious disease and drug resistance, such as the case of malaria, the movements of women are often not as clearly captured in models as those of males who work as farmers or forestry workers. In the context of COVID‐19, studying changing mobility patterns by gender, age, race, and ethnicity, as well as other variables, would contribute important insights into behaviors and trends during the pandemic as well as possible contributing factors to patterns of infection.

### Land use and environmental change

How geographic entities change over time is fundamental to geographical analysis. Land system science posits land use as the interface between social and environmental systems, and as a central component of sustainability science (Müller and Munroe 2014). Spatial analysis of land systems, strongly informed by land rent and location theory, involves modeling the tradeoffs among competing land uses, like cattle ranching’s impact on Amazonian rainforest (Walker, 2014) or promoting mixed‐use development in Dhaka, Bangladesh (Sharmin and Haque 2019). Taking gender as an example, recent scholarship in land systems has emphasized that it is a mediating factor in land management, that spatial patterns of land use are not strictly reducible to profit‐ or utility‐maximization assumptions (Turner et al. 2020), and that natural resource use is shaped by social interactions over space and time (Sen and Nagendra 2019). Therefore, to understand pressing environmental problems and drivers of global change, such as transformations at extractive and agrarian frontiers, we must incorporate gender directly into our spatial analyses (de la Vega‐Leinert and Schönenberg 2020). Furthermore, a feminist and intersectional spatial imagination (Ducre 2018; Eaves and Al‐Hindi 2020) can focus geographical analysis of environmental change, hazards and vulnerability (Preston and Yuen 2011) with a lens of environmental justice and, indeed, climate justice (Orff 2020; Robinson 2018).

## Conclusions

The research agenda—and call to action—put forth in this commentary highlights several important themes. First, gaps and bias in data—whether from traditional, government agencies or novel, user‐generated, or “big” data sources, are likely greatest for the most marginalized members of society. Thus, if some individuals are disproportionately omitted from the sample, the results of the analysis will be biased. We add representativeness in data to the grand challenges of GIS and spatial analysis, alongside the modifiable areal unit problem (MAUP) and scale in space and time (Cheng and Adepeju 2014). Second, the foundational theories through which we understand societal issues from segregation to accessibility were largely founded on now‐outdated notions of households, family structures, and workers. An update to these theories that accounts for more contemporary notions of society is due. Finally, modeling efforts that fail to consider differences in how disparate groups travel, behave, interact, or make decisions, and continue to group all individuals together will never garner results capable of transforming social relations.

In addition, the lack of diversity in the field of quantitative geography, consistent with data science‐related disciplines more broadly, means that the perspectives of most under‐represented groups are largely missing—from data, from the algorithms being developed, and from the research questions being posed. For example, according to Tong and Murray (2012) only about 10 percent of 131 spatial optimization scholars who had obtained a Ph.D. in geography or were with a geography program identified as female; this is much lower than the average female faculty (37 percent) in university and college geography departments (AAG 2015), suggesting that female scholars in the field of spatial optimization are seriously underrepresented. Similarly, as in the wider computer science community, the GeoAI community faces an inequality and lack of diversity crisis (West et al. 2019). Parallel discussions have arisen in ecology and evolution, as well (Mori, 2022). Diversifying our field will go a long way towards ensuring that research and subsequent policies, plans, and decisions affecting *everyone’s* life are addressed.

There is also an argument to be made for agitating against the principles of competition and ranking that govern the assessment of “quality” science, to say nothing of internet search engines. Perhaps it is time to adopt the San Francisco Declaration on Research Assessment (https://sfdora.org), to measure progress by quality rather than quantity, and to fight against cognitive waste by moving from a publish or perish mentality to one of collective survival (Kosmopoulos and Pumain 2007).

*Geographical Analysis*—and indeed all journals in the field—have a role to play in moving the state of knowledge and research forward where representativeness and inclusiveness are concerned. To that end, we issue ourselves three challenges. The first is a continued increase in the diversity and inclusiveness of the journal’s leadership team. Although *Geographical Analysis* is already a leader where the share of women on the editorial board is concerned (Franklin et al. 2021), there remains much room for improvement on a range of dimensions, including geography, ethnicity, and race. Second, we aim to increase the diversity of authors and co‐authors publishing in the journal. We are a flagship journal in the field and publication in our pages provides visibility and audience. In addition, as highlighted above, spatial researchers from a variety of backgrounds bring fresh perspectives to longstanding topics such as accessibility, spatial interaction, land use, *et cetera*, which serves to further enrich and diversify our discipline and journal. Third, and finally, where the agenda presented above is concerned, we issue an open call for contributions in these areas, whether novel applications or new data or methodological approaches. The challenge of representative and inclusive geographical analysis is open to all, and we welcome this research at *Geographical Analysis*!

## Note

1 This work began as a collaboration on ideas about gender, women and female perspectives in early 2021. As it evolved, it became clearer that the subject should be opened up to argue the need for increased diversity not just in terms of gender, but in terms of the variety of voices and experiences needed in spatial research and in conversation.
